# Comparison of Quality of Life, Sexual Satisfaction and Marital Satisfaction between Fertile and Infertile Couples 

**DOI:** 10.22074/ijfs.2016.5045

**Published:** 2016-09-05

**Authors:** Seyedeh Zahra Masoumi, Maryam Garousian, Somayeh Khani, Seyedeh Reyhaneh Oliaei, Arezoo Shayan

**Affiliations:** 1 Students Research Center, School of Nursing and Midwifery, Hamadan University of Medical Sciences, Hamadan, Iran; 2 Fatemieh Hospital, Hamadan University of Medical Sciences, Hamadan, Iran; 3 Dentistry Faculty, Hamadan University of Medical Sciences, Hamadan, Iran; 4 Department of Midwifery, School of Nursing and Midwifery, Hamadan University of Medical Sciences, Hamadan, Iran

**Keywords:** Infertility, Quality of Life, Sexual Satisfaction, Marital Relationship

## Abstract

**Background:**

Fertility plays an important role in sexual and psychological function in families. Infertility can result in major emotional, social, and mental disorders, including a reduction in satisfaction with marital life and quality of life. The present study aimed to compare the quality of life and marital satisfaction and sexual satisfaction between fertile and infertile couples.

**Materials and Methods:**

This analytical cross-sectional study was conducted on 250 couples at the Fatemiyeh Educational Research Center affiliated to Hamadan University of Medical Sciences, Hamadan, Iran, from May to August in 2014. The subjects were randomly selected from the patients referred to this center using a table of random numbers. They were then allocated into two groups of infertile group (n=125) and fertile group (n=125). The study participants completed World Health Organization Quality of Life-BREF (WHOQOL-BREF) questionnaire, Linda Berg’s Sexual Satisfaction Scale, and Enrich Marital Satisfaction Scale. Then, the data were entered into the SPSS version16 for statistical analysis. The Chi-square and Mann-Whitney tests were also applied to compare the data between the groups.

**Results:**

The results revealed no significant difference between the two groups regarding demographic and general health variables. The mean scores of sexual satisfaction were 63.67 ± 13.13 and 46.37 ± 7.72 in the fertile and infertile couples, respectively. Furthermore, the mean scores of marital satisfaction were also 44.03 ± 9.36 and 36.20 ± 4.03 in the fertile and infertile groups, respectively. Our finding demonstrated that the fertile couples obtained significantly higher mean scores of quality of life as well as lower mean scores of sexual satisfaction and marital satisfaction as compared to the infertile ones (P<0.001).

**Conclusion:**

According to the results, the fertile couples obtained significantly higher quality of life and lower sexual satisfaction and marital satisfaction as compared to the infertile ones. Therefore, holding consultation programs and conducting more studies are necessary for improving the quality of life and promoting sexual and marital satisfaction in infertile couples.

## Introduction

Quality of life is a complex concept that is related to physical health, psychological status, level of independence, social relations, personal beliefs, and environmental factors (1). It is also affected by age, culture, sex, education level, social status, disease, and social environment (2). Infertility is among the difficult conditions affecting quality of life. It is also among the major medical problems whose rate has increased by 50% since 1955. To date, 10-15% of couples suffer from infertility (3). Infertility status and its related factors affect the quality of life through creating psychosocial stress, reduction of life satisfaction, increase of marital conflicts, and decrease of sexual satisfaction and marital satisfaction (4, 5).

In fact, infertility is considered as a personal and social problem affecting couple’s life and family’s performance, so exposes couples to mental pressure and various psychological disorders (6, 7). Infertility, as an emotional shock, can even have an impact on couples’ communication, occupational, and sexual skills. Overall, infertility, as a serious medical problem, can have destructive effects on the quality of life (8). Evidence has indicated that infertility is a destructive or painful experience that leads to more disappointment, prostration, and anger in infertile couples as compared to their fertile peers. Besides, infertile couples have disturbed relationships with their spouses, families, and friends that make them more vulnerable to psycho-emotional disorders, depression, anxiety, low self-confidence, and dissatisfaction that subsequently lead to low quality of life (9). Nevertheless, some researches have demonstrated that in case of cooperation and sharing responsibilities between couples, treatment procedures can increase their intimacy and improve the quality of their marital life (10). 

In a study by Nourani et al. (11) that was conducted at the Majidi Treatment Center, Tabriz, Iran, 12% of women reported low life quality, while more than half of them had desirable quality of life. In addition, familial and social pressure had a negative influence on infertile women’s quality of life. Marital and sexual satisfaction considerably affect couples’ physical and mental health. On the other hand, incompatibility in a marital relationship disturbs social relations, leads to tendency towards social deviations, and declines cultural values between couples. Thus, sexual satisfaction is necessary for solidity of marital life. Some researchers believe that sexual dissatisfaction accounts for 80% of marital conflicts (12). On the other hand, fertility status is one of the effective factors in sexual satisfaction. Based on various studies, infertility could result in several psychological disorders, including sexual dissatisfaction (13). However, some studies have shown no difference between the couples under infertility treatment and fertile couples in the context of marital satisfaction. In other words, marital satisfaction was not affected by infertility (14, 15). Tao in a systematic review has investigated marital relationship in infertility and reported that sexual satisfaction had impact on marital satisfaction (15). The problem of infertility has become deeper, especially in the Iranian culture in which there is extended family type. Because in this type of families having children is important. Infertility can be regarded as life crisis, identity crisis, chronic disease, or a combination of them (16). 

Quality of life in infertile couple differs from one society/culture to another. Fertility is of utmost sociocultural importance, while controversial results have been obtained regarding the effect of infertility on quality of life in western countries. Besides, only limited studies have been performed in this regard in the Eastern Counties, including Iran. Due to inconsistencies in the available studies, this study aimed to compare the quality of life, sexual satisfaction and marital satisfaction between fertile and infertile couples.

## Materials and Methods

The present analytical cross-sectional study was conducted on fertile couples and infertile couples referred to the Fatemieh Educational Research Center affiliated to Hamadan University of Medical Sciences, Hamadan, Iran, from May to August in 2014. The study protocol was approved by the Institutional Review Board and the Human Research Ethics Committee of the Hamadan University of Medical Sciences. Sample size was estimated using the following formula: 

$$n=\frac{{\left(Z_{1-\frac{\alpha }{2}}+Z_{1-\beta }\right)}^{2}\left(\sigma_{1}^{2}+\sigma_{2}^{2}\right)}{{\left({µ}_{2}-{µ}_{1}\right)}^{2}}$$

To achieve power of 90 and level of significance of 0.05, 125 couples were determined for each group.

At first, a list of eligible couples, referred to the Fertility Center of Hamadan University of Medical Sciences, was prepared, among whom 125 infer- tile couples were selected using a table of random numbers. Then, 125 fertile couples were selected from those referring to other clinics (like women’s health care, oncology, and children’s health care centers) using the same method, meaning 25 fertile couples were chosen from each clinic. The two groups in terms of age, socio-economic status and lack of acute or chronic diseases were matched.

The inclusion criteria of the infertile group were as follows: not conceiving after 5 years of trying, primary infertility, male factor infertility/female factor infertility (or both), unexplained infertility, and literacy skills in Farsi. The inclusion criteria for the fertile group were as follows: not suffering from infertility, having at least one child, willingness to cooperate in the study, and literacy skills in Farsi. On the other hand, the exclusion criteria of the study were as follows: use of medications other than those used for infertility treatment, physical or mental disorders, death of close relatives during the past two months, child adoption, and unwillingness to cooperate in the study . After the study objectives and procedures were explained to them, all participants signed a written informed consent.

### Measurement tools

All participants completed demographic questionnaire, World Health Organization Quality of Life-BREF (WHOQOL-BREF) questionnaire, Linda Berg’s Sexual Satisfaction Scale, and Enrich Marital Satisfaction Scale.

WHOQOL-BREF questionnaire prepared by the WHO contains 24 items regarding physical health (7 items), mental health (6 items), social relationships (3 items), and environmental health (8 items) dimensions. This questionnaire also includes 2 other items to evaluate health status and quality of life generally. Thus, the questionnaire has 26 items. The items are responded through a 5-option Likert scale and a score between 0 and 100 is assigned to each dimension (17). Assessment of psychometric properties was done by the WHO (18). In Iran, the reliability and validity of this scale were approved by Nejat et al. (19). Besides, Keramat et al. (20) have evaluated the reliability of WHOQOL-BREF and reported the Cronbach’s alpha (reliability) of 0.78, 0.77, and 0.79 belonging to physical health, mental health, and environmental health dimensions, respectively. 

Enrich Marital Satisfaction Scale designed by Olsun, Furnier, and Druckman contains 14 subscales. The reliability of this questionnaire was already confirmed (α=0.92), while Pazandeh and Sharghi (16) have reported a reliability of α=0.95. This scale is responded using a 5-option Likert scale and a score between 1 and 5 is allocated to each item. Accordingly, the scores are below 30 indicating severe dissatisfaction, between 30 and 40 indicating dissatisfaction, between 40 and 60 indicating relative and moderate satisfaction, between 60 and 70 indicating great satisfaction, and above 70 indicating very great satisfaction. Keramat et al. (20) have confirmed the reliability of this scale with Cronbach’s alpha of 0.91.

Linda Berg’s Sexual Satisfaction Scale designed by Linda Berg and Cresy in 1997 consists of 17 items with the following options: always, often, sometimes, rarely, and never receiving 5, 4, 3, 2, and 1 scores, respectively. Thus, the minimum and maximum scores of the scale are 17 and 85, respectively. Accordingly, the scores are 17-51 indicating weak, 52-67 indicating moderate, and 68-85 indicating good sexual satisfaction. The validity and the reliability (Cronbach’s alpha=0.94) of this scale were also approved by Keramat et al. (20). Assessment of psychometric properties of all questionnaire were done in many studies (18).

### Statistical analysis

All data analyses were performed using the Statistical Package for the Social Sciences (SPSS, SPSS Inc., USA) version 16. Chi-square test and correlation analysis were used to assess the relationship between the study variables. Besides, student’s t test was employed for comparison of the study groups. A value of P<0.05 was considered as statistically significant.

## Results

According to the results, the mean score of physical dimension of WHOQOL-BREF was significantly higher in the fertile group (15.46 ± 2.66) compared to the infertile group (14.86 ± 2.66, P<0.05). Also, in the environmental dimension, the fertile couples obtained a significantly higher mean score (13.90 ± 2.41) in comparison to the infertile ones (13.13 ± 2.49, P<0.05). In the mental dimension, the fertile group gained a higher mean score (13.71 ± 2.73) compared to the infertile group (13.42 ± 2.64), indicating the difference was not statistically significant. Considering the social dimension, although no significant difference was observed between the two groups, the mean score of the infertile group (14.27 ± 2.85) was higher than that of the fertile group (13.97 ± 2.85). 

The mean score of sexual satisfaction was significantly higher in the infertile group compared to the fertile group (63.67 ± 13.13 vs. 46.37 ± 7.72). The mean score of marital satisfaction was also significantly higher in the infertile couples compared to the fertile ones (44.03 ± 9.36 vs. 36.20 ± 4.03) (Table 1). The results showed weak sexual satisfaction in 21.6% of infertile women, 79.2% of fertile women, 15.2% of infertile men, and 62.4% of fertile men. In other words, weak sexual satisfaction was less common in infertile couples (Table 2). Moreover, relative and moderate marital satisfaction was observed among 48% of infertile women, 12% of fertile women, 52% of infertile men, and 9.6% of fertile men. Moreover, very great marital satisfaction was found neither in the infertile nor in the fertile group, and great satisfaction was also not observed in the fertile couples (0 vs. 32% in infertile women and 8.8% in infertile men). In other words, marital satisfaction was higher in the infertile couples compared to the fertile ones (Table 3).

**Table 1:** Comparison the means scores of different dimensions belonging to WHOQOL-BREF questionnaire between two groups using t test


Variables	Group	Frequency	Mean	SD	t	P value

Physical dimension	Fertile	250	15.46	2.66	2.56	0.01
	Infertile	250	14.86	2.66		
Mental dimension	Fertile	250	13.71	2.73	1.20	0.23
	Infertile	250	13.42	2.64		
Social dimension	Fertile	250	13.97	2.74	-1.20	0.231
	Infertile	250	14.27	2.85		
Environmental dimension	Fertile	250	13.90	2.41	3.50	<0.001
	Infertile	250	13.13	2.49		
Sexual satisfaction	Fertile	250	46.37	7.72	-17.96	<0.001
	Infertile	250	63.67	13.13		
Marital satisfaction	Fertile	250	36.20	4.03	-12.14	<0.001
	Infertile	250	44.03	9.36		


WHOQOL-BREF; World Health Organization Quality of Life-BREF questionnaire.

**Table 2:** Comparison of frequency distribution of different sexual satisfaction categories between fertile and infertile couples


Sexual satisfaction categories	Infertile couples n=125						Fertile couples n=125					
	Infertile women		Infertile men		Total		Fertile women		Fertile men		Total	
	n	%	n	%	n	%	n	%	n	%	n	%

Weak	27	21.6	19	15.2	46	36.8	99	79.2	78	62.4	177	141.6
Moderate	52	41.6	45	36.0	97	77.6	26	20.8	47	37.6	73	58.4
Good	46	36.8	61	48.8	107	85.6	0	0	0	0	0	0
Total	125	100	125	100	250	100	125	100	125	100	250	100


WHOQOL-BREF; World Health Organization Quality of Life-BREF questionnaire.

**Table 3:** Comparison of frequency distribution of marital satisfaction categories between fertile and infertile couples


Marital satisfaction categories	Infertile couples n=125						Fertile couples n=125					
	Infertile women		Infertile men		Total		Fertile women		Fertile men		Total	
	n	%	n	%	n	%	n	%	n	%	n	%

Severe dissatisfaction	7	5.6	5	4.0	12	9.6	14	11.2	4	3.2	18	14.4
Dissatisfaction	54	43.2	44	35.2	98	78.4	96	76.8	109	87.2	205	164
Relative and moderate satisfaction	60	48.0	65	52.0	125	100	15	12	12	9.6	27	21.6
Great satisfaction	4	32	11	8.8	15	40.8	0	0	0	0	0	0
Very great satisfaction	0	0	0	0	0	0	0	0	0	0	0	0
Total	125	100	125	100	250	100	125	100	125	100	250	100


## Discussion

Quality of life is the general well-being of individuals and societies, outlining negative and positive features of life. Sexual satisfaction is defined as an individual’s judgment about pleasure of one’s sexual behavior (21). The most important goal of sexual desire is reproduction and childbearing. Thus, sexual satisfaction is highly influenced by infertility (22). Compatibility and marital satisfaction are referred to a status in which a couple feels happy and satisfied that is created through mutual interest, caring, acceptance, understanding, and meeting each other’s needs, including sexual needs (23).

Throughout the recent two decades, quality of life has been considered as a major concern. In 1978, WHO indicated quality of life improves when an individual receives mental and physical care. Fertility has social, psychological and physiological aspects. In most cultures, reproduction is of great importance concept (24). The results of the present study showed that the fertile women obtained higher mean scores in all dimensions of quality of life compared to the infertile ones. This difference was statistically significant in physical and environmental dimensions, but not in mental and social dimensions. Infertility and other related issues, like treatment process, have a negative impact on physical and mental health of infertile couples.

Physical dimension (general health, physical role, and bodily pain) is a highly important aspect of life quality. Stress-related infertility leads to physiological stress that results in serious health problems (25). Infertile couples seeking treatment also experience a lot of physical problems (26). In current study, physical dimension of infertile couples is lower than fertile couples. In a study by Kamkary and Shokrzadeh (27), they have showed that control of the environmental factors, which is among psychological components, is higher in couples with higher mental functions. Mental pressures resulting from infertility affects couples’ attitude towards the environmental factors and reduces their determination to achieve their personal goals (28). According to the study by Direkvand Moghadam et al. (3), infertile women showed a lower mean score of physical role limitation due to physical problems as compared to the fertile ones. In addition, in a study by Hatamloye Saedabadi and Hashemi Nosratabad (29), he has indicated that control over the environment was lower among infertile women compared to fertile women. These results were consistent with those of the current study, showing that environment dimension of the quality of life in infertile couples was lower than fertile couples. 

Evidence has demonstrated that psychological stress due to infertility treatment affect patients’ quality of life through disturbing their psychological, social, and welfare conditions (30). Infertility is a source of social pressure that is exerted by a traditional culture surrounding the infertile couples (31). A study has shown that infertile couples have more feelings of helplessness and disappointment (32). One study has revealed that almost one thirds of all women and their partners experienced a lack of social support (30). Nevertheless, some researchers have reported higher social support among infertile women. A study performed in Turkey has also showed that in spite of lower scores in mental dimension, infertile women had better social support (33). In our study, no significant difference was also found between fertile and infertile couples regarding mental and social dimensions. Unlike, another study in Iran has revealed statistically significant relationship between duration of infertility and mental disorders and marital conflicts (34). The findings of the current study demonstrated significantly higher sexual satisfaction among the infertile couples compared to the fertile ones, indicating the couples may be closer emotionally and psychologically to each other because of the conditions and continuation of treatment.

A previous study has indicated that infertile couples had lower sexual function in orgasm, arousal, and desire dimensions (23). On the other hand, a study by Jamali et al. (22) conducted in Iran has showed that infertility had no impact on couples’ sexual function. Also, another study has indicated that sexual dysfunction was only detected in 11% of infertile couples (35). Similarly, Monga et al. (36) has reported no significant difference between infertile and fertile couples with respect to sexual function, which was attributed to the need for large number of sexual relationships for treatment of infertility and getting pregnant. Yet, this can also be associated with an increase in intimacy of infertile couples (22). In the present study, the infertile couples showed higher marital satisfaction compared to the fertile ones. In a study by Lotfi Kashani and Vaziri (37), they have concluded that marital satisfaction was accompanied by sexual satisfaction, which in return, higher sexual satisfaction resulted in higher marital satisfaction. Up to now, a large number of researches have shown that infertility declined marital satisfaction (38). However, many studies have indicated that children play a major role in decreasing marital satisfaction (39). Although having children strengthens the marital relationship, it may decrease marital satisfaction with passage of time and growing number of children (40, 41). Overall, the findings of our study showed that despite a decrease in life quality, infertile couples had high sexual and marital satisfaction. There were a number of limitations in this study. Due to randomly selected samples, there was no choice to select all samples properly. Therefore, it is recommended that in future, a study should be conducted with a larger sample size to show the improvement of quality of life and marital satisfaction of infertile couples as the basis of family and society.

## Conclusion

According to the results, the fertile couples obtained significantly higher quality of life and lower sexual satisfaction and marital satisfaction as compared to the infertile ones. This might have resulted from disturbance in couples’ marital relationships due to children, financial problems, etc. Therefore, holding consultation programs and conducting more studies are necessary for improving the quality of life and promoting sexual satisfaction and marital satisfaction in infertile couples. 
